# Association of triglyceride-glucose index and estimated glucose disposal rate with outcomes in patients with acute myocardial infarction: Cumulative effect and mediation analysis

**DOI:** 10.1371/journal.pone.0328150

**Published:** 2025-07-23

**Authors:** Yazhao Sun, Pei Sun, Dongsheng Liu

**Affiliations:** Department of Cardiology, Cangzhou People’s Hospital, Cangzhou, Hebei Province, China; Ferdowsi University of Mashhad, IRAN, ISLAMIC REPUBLIC OF

## Abstract

**Background:**

The triglyceride-glucose (TyG) index and the metabolic score for insulin resistance (METS-IR) are insulin resistance indicators based on different metabolic parameters. However, their cumulative effect on the outcomes of patients with acute myocardial infarction (AMI) remains unclear. This study aims to investigate whether the combined assessment of the TyG index and METS-IR can improve risk stratification and prognostic prediction in AMI patients.

**Methods:**

This retrospective cohort study included AMI patients admitted to Cangzhou People’s Hospital from January to December 2018. The baseline TyG index and METS-IR were calculated for each patient. The primary endpoint was major adverse cardiovascular and cerebrovascular events (MACCEs) during a 6-year follow-up, defined as a composite of all-cause mortality, coronary revascularization, and stroke. Logistic regression models and restricted cubic splines (RCS) were used to assess the association between TyG index, METS-IR, and the risk of MACCEs. Receiver operating characteristic (ROC) curves were applied to evaluate the discriminative ability of TyG index, METS-IR, and their combined predictive model (TyG index + BMI) for MACCEs. The area under the curve (AUC) was calculated to quantify predictive performance. Additionally, the net reclassification index (NRI) and integrated discrimination improvement (IDI) were computed to assess the incremental predictive value of TyG index + METS-IR beyond traditional risk factors. Subgroup analyses were conducted, and mediation analysis was performed to explore the potential mediating role of METS-IR in the relationship between TyG index and MACCEs.

**Results:**

A total of 1,899 patients were included in the study. Multivariable logistic regression analysis showed that TyG index (OR = 1.655, 95% CI: 1.305–2.100, *P* < 0.001) and METS-IR (OR = 1.026, 95% CI: 1.001–1.052, *P* = 0.048) were both independent risk factors for MACCEs. Further analysis showed that patients with both high TyG index and high METS-IR had the highest risk of MACCEs (OR = 1.908, 95% CI: 1.188–3.114, *P* = 0.008). ROC curve analysis demonstrated that the combined prediction of MACCEs using TyG index and METS-IR achieved an AUC of 0.625, which was significantly superior to METS-IR alone (AUC = 0.573, *P*_DeLong_ = 0.003). When compared with the traditional risk prediction model (AUC = 0.696), incorporating TyG index and METS-IR significantly improved predictive performance (optimized AUC = 0.717, P _DeLong_ = 0.038). This also resulted in notable enhancements in NRI (0.353, *P *< 0.001) and IDI (0.156, *P* < 0.001). Subgroup analysis revealed no significant interaction effects of sex, age, hypertension, or diabetes status on the association between TyG index, METS-IR, and MACCEs (*P*-interaction > 0.05). Mediation analysis indicated that METS-IR partially mediated the relationship between TyG index and MACCEs.

**Conclusion:**

TyG index and METS-IR are predictors of adverse outcomes in AMI patients.

## 1 Introduction

Acute myocardial infarction (AMI) is one of the leading causes of mortality and morbidity worldwide, with its prognosis closely linked to metabolic disorders [1,2]. Despite continuous improvements in treatment strategies in recent years, there remains significant heterogeneity in the long-term prognosis of AMI patients. Accurately identifying high-risk patients and optimizing risk stratification remain key challenges in clinical practice. Insulin resistance (IR) plays a critical role in the development of atherosclerosis and myocardial injury repair. Its mechanisms include endothelial dysfunction, chronic inflammation, and oxidative stress [3,4]. However, traditional IR assessment methods, such as the hyperinsulinemic-euglycemic clamp technique, are considered the “gold standard” but are difficult to implement in clinical settings due to their complexity and high cost [5]. Therefore, finding simple and reliable alternative indicators of IR has become a focus of recent research.

The triglyceride-glucose (TyG) index and the metabolic score for insulin resistance (METS-IR) are alternative IR indicators developed in recent years based on routine metabolic parameters [6,7]. These indices more accurately reflect the core mechanisms of IR, namely glucotoxicity and lipotoxicity. The TyG index combines fasting triglycerides (TG) and fasting plasma glucose (FPG) levels, effectively reflecting the synergistic effects of lipid metabolism disorders and glucose homeostasis imbalance [6]. Numerous studies have demonstrated that the TyG index is significantly associated with an increased risk of cardiovascular diseases (CVDs), such as arterial stiffness, coronary artery disease, and hypertension [8–10]. The TyG index has proven to be a reliable and convenient prognostic marker for adverse outcomes in patients with acute decompensated heart failure, acute coronary syndrome, and severe cerebrovascular diseases [11–13]. In addition, the TyG index is strongly correlated with the incidence of several diseases, including diabetes, fatty liver, cancer, kidney disease, and disorders of the reproductive system [14,15]. METS-IR integrates body mass index (BMI), FPG, TG, and the logarithmic transformation of high-density lipoprotein cholesterol (HDL-C), offering a comprehensive assessment of IR from the perspectives of obesity and metabolic syndrome [7]. Researchers have observed an association between METS-IR and diseases such as CVD, hypertension, stroke, obstructive sleep apnea, and hyperuricemia [16–20]. Notably, METS-IR has been shown to be more accurate than the TyG index in predicting all-cause mortality and cardiovascular mortality [21].

However, research on the value of the TyG index and METS-IR in predicting AMI prognosis, and whether there is an interaction between the two, remains limited. Therefore, this study aims to explore the independent and combined predictive performance of the TyG index and METS-IR in AMI patients. It will also evaluate their relationship with major adverse cardiovascular and cerebrovascular events (MACCEs). The findings are expected to provide new evidence for metabolic risk assessment in AMI patients and offer references for precise risk stratification and personalized management strategies.

## 2 Methods

### 2.1 Study population

This retrospective cohort study consecutively included 2,746 AMI patients admitted to Cangzhou People’s Hospital from January to December 2018, all of whom received a drug-eluting stent during their hospitalization. Patients were excluded if they had a history of myocardial infarction (MI) (N = 36), missing key metabolic data (such as FPG, TG, HDL-C, or BMI, affecting the calculation of TyG and METS-IR) (N = 368), malignant tumors (N = 21), severe hepatic dysfunction (alanine aminotransferase > 5 times the upper limit of normal) (N = 72), renal insufficiency (serum creatinine ≥1.5 mg/dL or estimated glomerular filtration rate < 30 mL/min/1.73m²) (N = 143), or missing follow-up data (N = 207). A total of 1,899 patients were included in the final analysis. The study was approved by the Ethics Committee of Cangzhou People’s Hospital and the Institutional Review Board (Approval No.: K2024-094–01). All patients provided written informed consent for the review of their medical records and participation in the study. The research was conducted in accordance with the ethical standards of the Declaration of Helsinki, ensuring the rights, safety, and well-being of all participants were protected throughout the study.

### 2.2 Data collection

Baseline data of participants were collected through the hospital’s electronic medical record system and standardized baseline surveys, covering information related to sociodemographic status and health-related factors, including age, gender, and BMI. BMI = weight (kg)/ [height (m)] ². Health-related factors include self-reported smoking status (current or former smoker, never smoked), self-reported physician-diagnosed medical conditions (such as percutaneous coronary intervention [PCI], stroke, hypertension, diabetes, atrial fibrillation, and heart failure), and whether diagnosed with ST-elevation myocardial infarction (STEMI). Laboratory markers include white blood cell, hemoglobin, platelet, uric acid, and creatinine. Fasting markers (after at least 8 hours of fasting) include FPG, TG, HDL-C, total cholesterol (TC), and low-density lipoprotein cholesterol (LDL-C).The TyG was calculated using the formula: TyG = Ln [TG (mg/dL) × FPG (mg/dL)/ 2]. The METS-IR index was calculated using the formula: METS-IR = Ln (FPG [mg/dL] × 2 + TG [mg/dL]) × BMI/ Ln (HDL-C [mg/dL]). Lesion characteristics, including the number of stents, number of diseased vessels, and number of treated vessels, as well as medications at discharge, including antiplatelet drugs, lipid-lowering agents, and antidiabetic medications.

### 2.3 Study endpoints

All patients were followed up for six years. The follow-up assessments were conducted at 3 months, 6 months, 12 months, and annually thereafter.The primary endpoint of this study was defined as MACCEs, which included a composite of all-cause mortality, coronary revascularization, and stroke. Follow-up data were obtained through outpatient visits, telephone interviews, or hospitalization records.

### 2.4 Statistical analyses

All statistical analyses were performed using R statistical software (R Foundation for Statistical Computing, Vienna, Austria, version 4.3.1). Data were described as means and standard deviations (SDs) for normally distributed continuous variables, and as medians and interquartile ranges for nonnormally distributed continuous variables. Frequency with percentage was used to describe categorical variables. The study population was divided into two groups based on the occurrence of MACCEs, and baseline characteristics were compared between groups using the χ² test, Student’s t-test, or Mann-Whitney U test, as appropriate.

To explore the association between TyG index, METS-IR, and MACCEs, we constructed two logistic regression models. TyG index and METS-IR were analyzed as both continuous and categorical variables. When treated as categorical variables, the study population was divided into four groups based on the median values (TyG index = 8.935, METS-IR = 40.673): low TyG index and low METS-IR (reference group), high TyG index and low METS-IR, low TyG index and high METS-IR, and high TyG index and high METS-IR. Model 1 was unadjusted, while Model 2 was further adjusted for prior stroke, diabetes, atrial fibrillation, heart failure, creatinine, TC, LDL-C, number of stents, number of diseased vessels, and number of treated vessels, in addition to including TyG index and METS-IR. The results were presented as odds ratios (ORs) with 95% confidence intervals (CIs). Furthermore, to investigate the potential dose-response relationship between TyG index, METS-IR, and MACCEs, we incorporated restricted cubic splines (RCS) into the logistic regression model for analysis.

We used receiver operating characteristic (ROC) curves to evaluate the discriminatory ability of TyG index, METS-IR, and their combined prediction model (TyG index + METS-IR), and calculated the area under the curve (AUC) to assess their predictive performance. Additionally, to further evaluate the incremental predictive value of TyG index + METS-IR over traditional risk factors, we calculated the net reclassification index (NRI) and the integrated discrimination improvement (IDI). Furthermore, subgroup analyses were performed based on age, sex, hypertension, and diabetes subtypes. We also conducted a mediation analysis to assess the mediating effect of METS-IR in the relationship between TyG index and MACCEs. The indirect effect mediated by METS-IR, the direct effect not mediated by METS-IR, and the proportion mediated were calculated and reported. We considered two sided *P* values < 0.05 to be significant.

## 3 Results

### 3.1 Participant characteristics

A total of 1,899 patients were included in this study, and their baseline characteristics were compared based on the occurrence of MACCEs (Table 1). The results showed that, compared to individuals without MACCEs, patients who experienced MACCEs were more likely to have a history of stroke, diabetes, atrial fibrillation, and heart failure. Additionally, they had a higher proportion of stent implantation ≥2, diseased vessels ≥2, and treated vessels ≥2. In terms of laboratory parameters, patients in the MACCEs group exhibited significantly higher levels of creatinine, TC, and LDL-C. Moreover, both TyG index and METS-IR were at higher levels in this group.

**Table 1:** Baseline characteristics of non-MACCE and MACCE participants.

Characteristic	Overall (n = 1899)	Non-MACCEs (n = 1715)	MACCEs (n = 184)	*P*-value
Demographic Characteristics				
Age, years	63.00 (54.00 - 69.00)	63.00 (54.00 - 69.00)	64.00 (57.00 - 70.00)	0.192
Male, n	1,269.00 (66.82%)	1,145.00 (66.76%)	124.00 (67.39%)	0.864
BMI, kg/m²	25.39 (23.05 - 27.68)	25.39 (23.03 - 27.68)	25.42 (23.27 - 27.59)	0.812
Smoking History, n	925.00 (48.71%)	838.00 (48.86%)	87.00 (47.28%)	0.684
Medical History, n				
Prior PCI	188.00 (9.90%)	164.00 (9.56%)	24.00 (13.04%)	0.133
Prior Stroke	206.00 (10.85%)	178.00 (10.38%)	28.00 (15.22%)	0.045
Hypertension	1,013.00 (53.34%)	910.00 (53.06%)	103.00 (55.98%)	0.451
Diabetes	443.00 (23.33%)	384.00 (22.39%)	59.00 (32.07%)	0.003
Atrial Fibrillation	69.00 (3.63%)	56.00 (3.27%)	13.00 (7.07%)	0.009
Heart Failure	254.00 (13.38%)	217.00 (12.65%)	37.00 (20.11%)	0.005
Clinical Presentation, n				
STEMI	899.00 (47.34%)	807.00 (47.06%)	92.00 (50.00%)	0.447
Laboratory Data				
White Blood Cell, 109/L	8.84 (7.02 - 11.16)	8.82 (7.02 - 11.14)	9.10 (7.06 - 11.18)	0.468
Hemoglobin, g/L	139.66 ± 15.77	139.58 ± 15.74	140.45 ± 16.02	0.484
Platelet, 109/L	217.00 (180.00 - 256.00)	216.00 (180.00 - 256.00)	221.00 (180.00 - 257.00)	0.621
Uric Acid, µmol/L	301.00 (249.00 - 362.00)	301.00 (249.00 - 361.50)	305.00 (253.00 - 372.50)	0.225
Creatinine, µmol/L	65.00 (55.00 - 77.00)	65.00 (54.70 - 76.80)	68.50 (57.00 - 83.50)	<0.001
FPG, mg/dL	120.71 (100.35 - 156.74)	119.63 (99.45 - 153.50)	136.02 (108.19 - 193.76)	<0.001
ALT, U/L	23.00 (16.00 - 36.40)	23.00 (16.00 - 37.00)	23.00 (16.00 - 35.00)	0.956
TC, mg/dL	177.17 (151.70 - 203.42)	175.63 (151.31 - 201.11)	185.09 (158.07 - 219.25)	<0.001
TG, mg/dL	121.25 (85.85 - 179.66)	121.25 (84.96 - 177.89)	134.08 (87.62 - 191.16)	0.073
HDL-C, mg/dL	41.69 (35.51 - 49.79)	41.69 (35.51 - 49.79)	42.07 (35.13 - 49.22)	0.813
LDL-C, mg/dL	108.08 (85.69 - 130.47)	106.92 (85.69 - 129.31)	115.80 (90.71 - 141.86)	0.002
TyG index	8.93 (8.50 - 9.46)	8.91 (8.47 - 9.43)	9.15 (8.73 - 9.69)	<0.001
METS-IR	41.03 ± 6.92	40.78 ± 6.63	43.39 ± 8.92	<0.001
Lesion Characteristics and Intervention, n				
Number of Stents ≥2	700.00 (36.86%)	606.00 (35.34%)	94.00 (51.09%)	<0.001
Number of Diseased Vessels ≥2	1,199.00 (63.14%)	1,062.00 (61.92%)	137.00 (74.46%)	<0.001
Number of Treated Vessels ≥2	504.00 (26.54%)	436.00 (25.42%)	68.00 (36.96%)	<0.001
Medications at Discharge, n				
Antidiabetic (Proportion of Diabetic Patients)	428.00 (96.61%)	369.00 (96.09%)	59.00 (100.00%)	0.122
Aspirin	1,881.00 (99.05%)	1,698.00 (99.01%)	183.00 (99.46%)	0.551
Clopidogrel/Ticagrelor	1,845.00 (97.16%)	1,667.00 (97.20%)	178.00 (96.74%)	0.720
β-Blockers	1,152.00 (60.66%)	1,038.00 (60.52%)	114.00 (61.96%)	0.706
ACEIs	1,128.00 (59.40%)	1,010.00 (58.89%)	118.00 (64.13%)	0.169
Statins	1,848.00 (97.31%)	1,671.00 (97.43%)	177.00 (96.20%)	0.323

**Abbreviations:** MACCEs, Major Adverse Cardiovascular and Cerebrovascular Events; BMI, Body Mass Index; STEMI, ST-Elevation Myocardial Infarction; FPG, Fasting Blood Glucose; ALT, Alanine Aminotransferase; TC, Total Cholesterol; TG, Triglycerides; HDL-C, High-Density Lipoprotein Cholesterol; LDL-C, Low-Density Lipoprotein Cholesterol; TyG, triglyceride-glucose; METS-IR, Metabolic Syndrome Insulin Resistance.

### 3.2 Association between TyG index, METS-IR, and MACCEs

**Fig 1:**
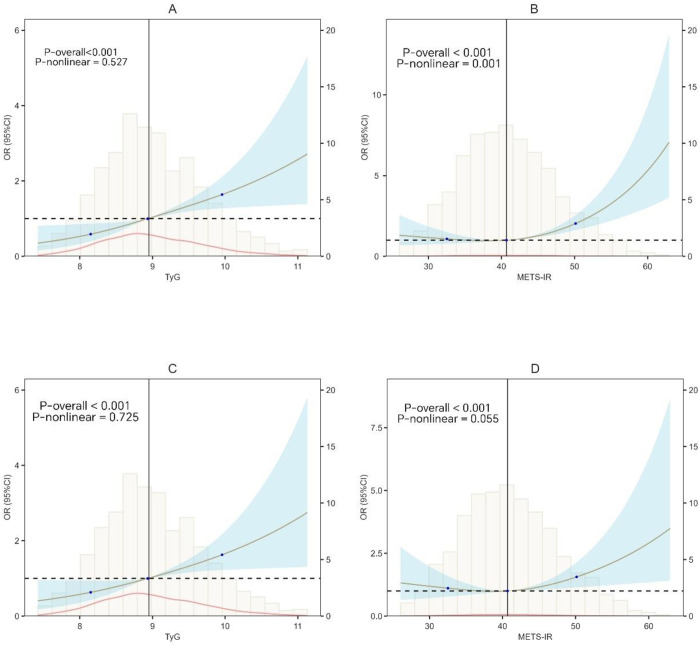
Restricted cubic spline curves of TyG and METS-IR for MACCEs. **(A)** Association Between TyG index and MACCEs (unadjusted). **(C)** Association Between TyG index and MACCEs (adjusted for prior stroke, diabetes, atrial fibrillation, heart failure, creatinine, TC, LDL-C, METS-IR, number of stents, number of diseased vessels, and number of treated vessels). **(B)** Association Between METS-IR index and MACCEs (unadjusted). **(D)** Association Between METS-IR index and MACCEs (adjusted for prior stroke, diabetes, atrial fibrillation, heart failure, creatinine, TC, LDL-C, TyG index, number of stents, number of diseased vessels, and number of treated vessels). Abbreviations: OR, Odds Ratio; CI, confidence interval; TyG, triglyceride-glucose; METS-IR, Metabolic Syndrome Insulin Resistance.

When TyG index and METS-IR were analyzed as continuous variables, the fully adjusted model indicated that both TyG index (OR = 1.655, 95% CI: 1.305–2.100, *P* < 0.001) and METS-IR (OR = 1.026, 95% CI: 1.001–1.052, *P* = 0.048) were independently associated with MACCEs in the overall population (Table 2). Further analysis using restricted cubic splines to assess the dose-response relationship between TyG index, METS-IR, and MACCEs revealed a linear trend (*P*-nonlinear > 0.05) (Fig 1).

**Table 2:** Association between TyG index, METS-IR, and MACCEs.

Continuous	Models	OR (95% CI)	*P*-value
Continuous Variable			
TyG index			
	Model 1	1.887 (1.281 - 2.801)	0.001
	Model 2	1.655 (1.305 - 2.100)	< 0.001
METS-IR			
	Model 1	1.046 (1.008 - 1.086)	0.018
	Model 2	1.026 (1.001 - 1.052)	0.048
Categorical Variable			
	Model 1		
Low TyG index and low METS-IR		(Reference)	
High TyG index and low METS-IR		1.631 (1.023 - 2.630)	0.041
Low TyG index and high METS-IR		1.281 (0.784 - 2.103)	0.324
High TyG index and high METS-IR		2.344 (1.523 - 3.678)	< 0.001
	Model 2		
Low TyG index and low METS-IR		(Reference)	
High TyG index and low METS-IR		1.626 (0.993 - 2.690)	0.055
Low TyG index and high METS-IR		1.209 (0.724 - 2.028)	0.468
High TyG index and high METS-IR		1.908 (1.188 - 3.114)	0.008

**Abbreviations:** OR, Odds Ratio; CI, confidence interval; TyG, triglyceride-glucose; METS-IR, Metabolic Syndrome Insulin Resistance.

Model 1 was unadjusted, while Model 2 included both TyG and METS-IR and was further adjusted for prior stroke, diabetes, atrial fibrillation, heart failure, creatinine, TC, LDL-C, number of stents, number of diseased vessels, and number of treated vessels.

When TyG index and METS-IR were analyzed as categorical variables based on their median values, the fully adjusted model showed that individuals in the high TyG index and high METS-IR group (TyG index > 8.935, METS-IR > 40.673) had the highest incidence of MACCEs (OR = 1.908, 95% CI: 1.188–3.114, *P* = 0.008) compared to the low TyG index and low METS-IR group (TyG index < 8.935, METS-IR < 40.673) (Table 2).

### 3.3 Predictive value of TyG index and METS-IR for MACCEs

**Fig 2:**
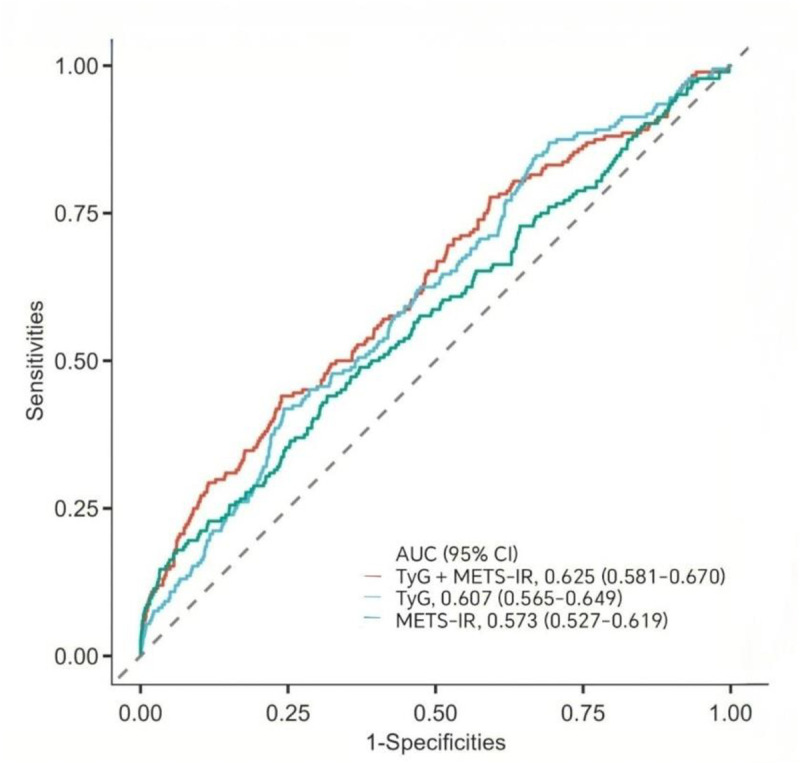
ROC Curves for Predicting MACCEs Using TyG index METS-IR and Individual Indicators. **+** Abbreviations: AUC, Area Under the Curve; CI, confidence interval; TyG, triglyceride-glucose; METS-IR, Metabolic Syndrome Insulin Resistance.

This study systematically evaluated the incremental value of the combined application of TyG index and METS-IR in predicting MACCEs. ROC curve analysis showed that the predictive performance of the combined TyG index and METS-IR model (AUC = 0.625) exhibited a slight improvement compared to TyG index alone (AUC = 0.607), although the difference was not statistically significant (*P*_DeLong_ = 0.239). However, compared to METS-IR alone (AUC = 0.573), the combined model demonstrated a significant advantage (*P*_DeLong_ = 0.003) (Fig 2).

**Fig 3:**
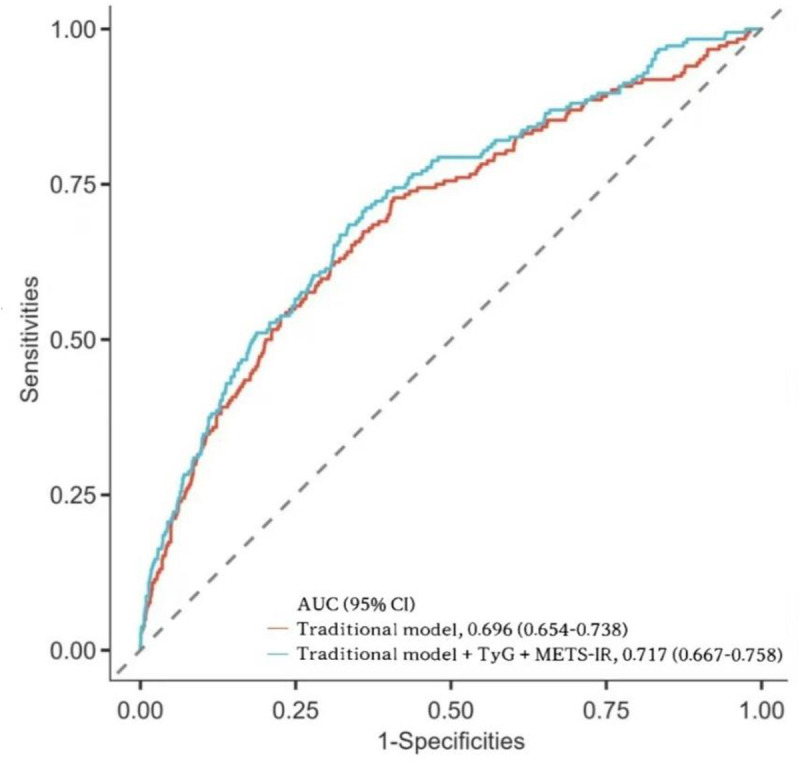
ROC Curves for Predicting MACCEs Using the Traditional Model and the Traditional Model + TyG index + METS-IR. Traditional model based on prior stroke, diabetes, atrial fibrillation, heart failure, creatinine, TC, LDL-C, number of stents, number of diseased vessels, and number of treated vessels. Abbreviations: AUC, Area Under the Curve; CI, confidence interval; TyG, triglyceride-glucose; METS-IR, Metabolic Syndrome Insulin Resistance.

More notably, compared with the traditional risk prediction model (baseline AUC = 0.696, 95% CI: 0.654–0.738), the inclusion of TyG index and METS-IR significantly enhanced the overall predictive ability of the model (optimized AUC = 0.717, 95% CI: 0.667–0.758, *P*_DeLong_ = 0.038) (Fig 3). Further analysis indicated that the incorporation of these combined indicators resulted in a significant improvement in integrated discrimination (IDI = 0.156, 95% CI: 0.008–0.024; *P* < 0.001) and a substantial net reclassification improvement (NRI = 0.353, 95% CI: 0.203–0.503; *P* < 0.001) (Table 3).

**Table 3:** Incremental predictive value of TyG index and METS-IR beyond traditional risk factors.

Models	NRI (95% CI)	*P* NRI	IDI (95% CI)	*P* IDI
Traditional model	Reference		Reference	
Traditional model + TyG index + METS-IR	0.353 (0.203-0.503)	<0.001	0.156 (0.008-0.024)	<0.001

**Abbreviations:** CI, confidence interval; NRI, net reclassification index; IDI, integrated discrimination improvement; TyG, triglyceride-glucose; METS-IR, Metabolic Syndrome Insulin Resistance.

Traditional model based on prior stroke, diabetes, atrial fibrillation, heart failure, creatinine, TC, LDL-C, number of stents, number of diseased vessels, and number of treated vessels.

Abbreviations: AUC, Area Under the Curve; CI, confidence interval; TyG, triglyceride-glucose; METS-IR, Metabolic Syndrome Insulin Resistance.

### 3.4 Subgroup analysis

We further conducted an exploratory subgroup analysis by stratifying the study population based on sex, age, hypertension, and diabetes status to evaluate the predictive performance of TyG index and METS-IR in different populations (Table 4). The results showed that TyG index exhibited a significant predictive association in males (OR = 1.754, *P* < 0.001), the < 65-year-old group (OR = 1.492, *P *= 0.011), the ≥ 65-year-old group (OR = 2.043, *P* < 0.001), and individuals with hypertension (OR = 1.995, *P* < 0.001). Notably, TyG index maintained robust predictive value in both diabetic (OR = 2.096, *P* = 0.001) and non-diabetic populations (OR = 1.469, *P* = 0.010), suggesting its applicability across different glycemic statuses. In contrast, METS-IR did not reach statistical significance across the subgroups, indicating that its predictive value in this population may be limited. Multivariable interaction tests suggested that the association direction of both indicators with outcomes remained consistent across different subpopulations (*P*-interaction > 0.05).

**Table 4:** Association between TyG index, METS-IR, and MACCEs in different subgroups.

Continuous	Subgroup	Event/Total (%)	OR (95% CI)	*P*-value	*P*-interaction
TyG	Sex				0.262
	Male	124/1269 (9.77)	1.754 (1.323 - 2.325)	< 0.001	
	Female	60/630 (9.52)	1.456 (0.919 - 2.307)	0.110	
	Age				0.184
	< 65 years	98/1053 (9.31)	1.492 (1.097 - 2.028)	0.011	
	≥ 65 years	86/846 (10.17)	2.043 (1.367 - 3.053)	< 0.001	
	Diabetes				0.240
	Yes	59/443 (13.32)	2.096 (1.366 - 3.218)	0.001	
	No	125/1456 (8.59)	1.469 (1.098 - 1.967)	0.010	
	Hypertension				0.062
	Yes	103/1013 (10.17)	1.995 (1.421 - 2.800)	< 0.001	
	No	81/886 (9.14)	1.383 (0.985 - 1.943)	0.061	
METS-IR	Sex				0.923
	Male	124/1269 (9.77)	1.021 (0.991 - 1.053)	0.171	
	Female	60/630 (9.52)	1.039 (0.994 - 1.086)	0.091	
	Age				0.931
	< 65 years	98/1053 (9.31)	1.023 (0.990 - 1.057)	0.179	
	≥ 65 years	86/846 (10.17)	1.032 (0.991 - 1.074)	0.127	
	Diabetes				0.405
	Yes	59/443 (13.32)	1.013 (0.967 - 1.062)	0.578	
	No	125/1456 (8.59)	1.030 (1.000 - 1.061)	0.053	
	Hypertension				0.999
	Yes	103/1013 (10.17)	1.027 (0.992 - 1.062)	0.130	
	No	811/886 (9.14)	1.026 (0.989 - 1.066)	0.175	

**Abbreviations:** OR, Odds Ratio; CI, confidence interval; TyG, triglyceride-glucose; METS-IR, Metabolic Syndrome Insulin Resistance.

TyG and METS-IR were included in the model simultaneously, with adjustments for prior stroke, diabetes, atrial fibrillation, heart failure, creatinine, TC, LDL-C, number of stents, number of diseased vessels, and number of treated vessels.

### 3.5 Mediation analysis of METS-IR in the association between TyG index and MACCEs

Further mediation analysis was conducted to explore the role of METS-IR in mediating the relationship between TyG index and MACCEs (Table 5). The results showed that the total effect was 0.043 (95% CI: 0.023–0.064, *P* < 0.001), indicating a significant impact of TyG index on MACCEs. The mediation effect was 0.002 (95% CI: 0.000–0.005, *P *= 0.048), suggesting that METS-IR played a partial mediating role in the association between TyG index and MACCEs, although the effect was relatively small. The direct effect was 0.041 (95% CI: 0.021–0.062, *P* < 0.001), demonstrating that the direct impact of TyG index on MACCEs remained dominant. The proportion mediated was 4.73%.

**Table 5:** Decomposition of the Total Association Between TyG index and MACCEs Into Direct and Indirect Associations Mediated by METS-IR.

Mediation effect	Estimate	95% CI lower	95% CI upper	*P*-value
Total effect	0.043	0.023	0.064	< 0.001
Mediation effect	0.002	0.000	0.005	0.048
Direct effect	0.041	0.021	0.062	< 0.001
Proportion mediated	0.047	0.001	0.133	0.048

**Abbreviations:** CI, confidence interval.

Adjusted for prior stroke, diabetes, atrial fibrillation, heart failure, creatinine, TC, LDL-C, number of stents, number of diseased vessels, and number of treated vessels.

## 4 Discussion

This study explored the association between the TyG index and METS-IR with the risk of MACCEs in AMI patients. The main findings are as follows: (1) During the 6-year follow-up, after adjusting for confounding factors in the logistic regression model, both the TyG index and METS-IR were independently associated with adverse outcomes, and no non-linear relationship was found between either of them and the risk of composite cardiovascular events. (2) Both the TyG index and METS-IR exhibited a potential cumulative effect on the risk of adverse outcomes. The combination of TyG index > 8.935 and METS-IR > 40.673 effectively identified the highest-risk individuals among AMI patients. (3) The AUC for the combined prediction of MACCEs using the TyG index and METS-IR was 0.625, which showed a significant advantage over METS-IR alone, but there was no statistically significant difference compared to TyG index alone. Furthermore, incorporating the TyG index and METS-IR into the traditional risk prediction model significantly improved predictive performance, with notable improvements in both the net reclassification index (NRI = 0.353, *P* < 0.001) and integrated discrimination improvement (IDI = 0.156, *P *< 0.001). (4) Gender, age, hypertension, and diabetes status did not significantly interact with the relationship between the TyG index, METS-IR, and MACCEs, suggesting that these two metabolic indicators have stable predictive value across different populations. (5) METS-IR partially mediated the relationship between the TyG index and MACCEs, indicating that METS-IR may play an important role in the link between metabolic disorders and adverse cardiovascular outcomes. The TyG index and METS-IR are practical and reliable tools for assessing cardiovascular risk in daily clinical practice. The TyG index, calculated using FPG and TG, is easy to measure and provides a quick screening method for identifying metabolic abnormalities and early cardiovascular risks, especially in primary care settings. METS-IR, which incorporates BMI, TG, FPG, and HDL-C, offers a more comprehensive evaluation of IR and metabolic dysfunction, helping clinicians to assess overall cardiovascular risk more accurately. Together, these indices enhance risk stratification, especially in patients at high risk for AMI. Their combination improves predictive performance and can be easily integrated into existing risk prediction models, offering clinicians a cost-effective, straightforward approach to identify high-risk patients and tailor prevention strategies accordingly.

IR refers to a condition in which the body’s biological response to a given concentration of insulin is lower than normal under steady-state conditions, indicating a decreased sensitivity of insulin target tissues to insulin [22]. The TyG index, as a metabolic-related indicator, reflects the degree of IR and lipid metabolism abnormalities. A study on the U.S. population with metabolic syndrome found that individuals in the higher quartiles of the TyG index had a higher risk of all-cause mortality and diabetes-related mortality [23]. In addition, the TyG index is closely associated with various cardiovascular and metabolic diseases, including sarcopenia, hyperuricemia, chronic kidney disease, hypothyroidism, and metabolic dysfunction-related fatty liver disease [24–28]. Recent research has shown that the TyG index, as a marker of atherosclerosis, is closely linked to the risk of various types of CVDs [29]. A cohort study involving 15,450 Japanese participants demonstrated that a higher TyG index was significantly associated with an increased risk of hypertension and prehypertension [30]. Tian et al. showed that both baseline and long-term elevated TyG index levels were associated with an increased risk of MI [31]. A study conducted in the Chinese population found that compared with the Q1 group of the TyG index, the Q4 group was significantly associated with moderate stroke risk (OR = 2.73; 95% CI: 2.50–2.99; *P* < 0.001), and was also significantly associated with high-risk stroke occurrence (OR = 5.39; 95% CI: 4.83–6.01; *P *< 0.001) [32].

IR is an independent predictor of CVDs, and its potential mechanisms are as follows: First, IR can induce compensatory hyperinsulinemia, leading to increased lipid synthesis, which in turn causes lipid metabolism disorders, including elevated TG, increased small dense low-density lipoprotein particles, postprandial lipidemia, and decreased HDL levels. These changes may promote the development of atherosclerosis [33]. As β-cell function in the pancreas gradually declines, hyperglycemia progresses to diabetes, which is also recognized as an important risk factor for atherosclerosis. Secondly, when blood glucose increases, the expression of angiotensinogen, angiotensin-converting enzyme, and angiotensin II significantly increases [34]. Meanwhile, compensatory hyperinsulinemia may activate the renin-angiotensin-aldosterone system, which could promote an increase in blood pressure [35]. Lastly, IR is closely associated with endothelial dysfunction, oxidative stress, and systemic metabolic inflammatory responses. These factors work together to increase the risk of CVDs [36,37]. Li et al. measured endothelial dysfunction using flow-mediated dilation and found that subjects with elevated TyG index were more likely to develop endothelial dysfunction [38].

Pu et al. analyzed data from the MIMIC-IV 3.0 database and found that in ischemic stroke patients, a higher TyG index was significantly associated with increased mortality risk at all time points, with patients in the highest TyG quartile exhibiting the highest risk [39]. Additionally, in patients with diagnosed ischemic cardiomyopathy, reduced insulin activity may limit glucose bioavailability, leading to a metabolic shift toward fatty acid oxidation. This metabolic change may increase myocardial oxygen consumption and reduce the compensatory capacity of unaffected myocardium [40]. Jia et al. found that IR was inversely associated with median endothelial colony-forming units, leading to a reduction in collateral vessel density during cardiac ischemic response [41]. Wang et al. demonstrated that the TyG index is a reliable marker for predicting coronary artery lesion severity and prognosis in acute coronary syndrome patients [42]. A meta-analysis showed that a higher TyG index can serve as an independent predictor for MACCEs and all-cause mortality risk in AMI patients [43]. Furthermore, a cohort study involving 776 type 2 diabetes patients who underwent PCI showed that the TyG index was positively correlated with future recurrent cardiovascular events, defined as all-cause mortality, non-fatal stroke, non-fatal myocardial infarction, or unplanned repeat revascularization [44]. Notably, a previous study by Francesca et al. indicated that the TyG index outperforms the homeostasis model assessment-insulin resistance (HOMA-IR) index in predicting MACCEs in acute coronary syndrome patients [45].

Research has found that a higher TyG index is associated with an increased risk of in-stent restenosis [46]. IR may not only lead to platelet hyperactivity, increasing the risk of thrombosis, but also promote the expression of tissue factor dependent on platelet-derived TxA2, further exacerbating thrombus formation [47]. IR is commonly accompanied by hyperglycemia, and excessive glycation processes promote the proliferation of smooth muscle cells, collagen crosslinking, and deposition, leading to cardiac fibrosis. These pathological changes result in diastolic stiffness of the left ventricle, which may ultimately lead to heart failure [48]. Li and colleagues observed that in a cohort of 1,028 obese patients, an increase in the TyG index was independently associated with subclinical left ventricular systolic dysfunction [49].

METS-IR, as a novel scoring system for screening insulin sensitivity, significantly improves the specificity of IR assessment by integrating body fat accumulation reflected by BMI, glucose-lipid metabolism disorders, and the inverse regulatory effect of HDL-C. This allows for effective identification of high-risk individuals with pathological changes associated with IR. METS-IR is significantly correlated with fat content in the liver and pancreas, and ectopic fat accumulation in muscle and liver tissue is considered a mechanism for the development of IR [50–52]. Previous studies have shown that METS-IR is positively correlated with an increased risk of CVDs [53–56]. METS-IR is also a reliable prognostic predictor for MACCEs in patients with early-onset coronary artery disease [57]. This study found that the TyG index and METS-IR have a significant cumulative effect on the prognosis of AMI patients. When both of these indices are elevated, patients face a higher risk of cardiovascular adverse events. The synergistic effect of TyG index and METS-IR may be amplified through the interaction of lipid metabolism disorders and visceral fat inflammation, thereby increasing the risk of cardiovascular diseases and related complications. Therefore, the combined assessment of TyG index and METS-IR not only provides more precise prognostic evaluation but also reveals potential mechanisms underlying cardiovascular events. Future research should further explore the complementary roles of these two indices in CVDs risk stratification and evaluate whether intervening in insulin resistance can effectively reduce the occurrence of cardiovascular adverse events.

## 5 Strengths and limitations

This study has several significant strengths. First, the TyG index and METS-IR are simple to measure and cost-effective, making them particularly suitable for resource-limited environments and primary healthcare settings. They provide convenient metabolic risk assessment tools for clinicians and have strong predictive power in AMI patients. Second, the study employed a 6-year follow-up, validating the long-term prognostic value of these two indices in AMI patients. Finally, the study explored the cumulative and mediating effects of the TyG index and METS-IR, offering a deeper understanding of their mechanisms in AMI patients and providing new insights for the prevention and management of cardiovascular diseases.

However, there are several limitations. First, due to the small sample size and the fact that it was conducted at a single center, the generalizability of the results may be limited, and the findings may not be applicable to more diverse populations. Second, the retrospective design of the study prevents the establishment of causal relationships between the TyG index, METS-IR, and cardiovascular outcomes. Third, laboratory parameters were measured only once during the study period, without dynamic monitoring, which may not fully reflect the changes in these markers, potentially affecting the accuracy and representativeness of the results. Additionally, although multiple known confounding factors were considered, other unmeasured variables (such as lifestyle factors, treatment adherence, and the types of antidiabetic medications [e.g., metformin, insulin, GLP-1 receptor agonists]) may not have been fully assessed, potentially leading to residual confounding. Moreover, some MACCEs events were collected through telephone interviews, which may introduce reporting bias. Finally, the study did not include some established IR markers (such as HOMA-IR) and novel IR markers (such as the CHG index), and a comparison of these markers with the TyG and METS-IR could provide additional valuable information.

## 6 Conclusion

This finding suggests that both the TyG index and METS-IR are important predictive indicators for MACCEs in AMI patients. The results highlight the combined exposure effect of TyG index and METS-IR levels in cardiovascular events and recommend the joint assessment of TyG index and METS-IR for more precise cardiovascular risk stratification.
